# Sleep disturbances and sleep quality among individuals diagnosed with osteoarthritis: a systematic review and meta-analysis

**DOI:** 10.3389/fmed.2025.1653047

**Published:** 2025-11-19

**Authors:** Ansuman Panigrahi, Priyanka Sahu, Swati Sambita Mohanty, Rutuparna Sibani Dandsena, Jewel Ipsita Sahani, Sanghamitra Pati

**Affiliations:** ICMR-Regional Medical Research Centre, Bhubaneswar, Odisha, India

**Keywords:** sleep disturbances, sleep quality, osteoarthritis knee, osteoarthritis hip, systematic review

## Abstract

**Introduction:**

Osteoarthritis is a leading cause of chronic pain and reduced mobility, especially in weight-bearing joints such as the knees and hips. While physical limitations associated with knee and/or hip osteoarthritis (KHOA) are well-documented, increasing attention is being paid to its impact on sleep disturbances and overall sleep quality. Understanding the extent and nature of these sleep-related issues is essential for the holistic management of knee and/or hip osteoarthritis. This study aims to synthesize current evidence on sleep disturbances and sleep quality in individuals diagnosed with knee and/or hip osteoarthritis.

**Methods:**

A systematic review and meta-analysis were conducted following the Preferred Reporting Items for Systematic Reviews and Meta-Analyses (PRISMA) guidelines. Comprehensive searches were performed across multiple electronic databases, including Medline via PubMed, EMBASE, CINAHL via EBSCO, Scopus, Web of Science, and ScienceDirect. Additionally, gray literature was sourced through Google Scholar, ProQuest, and Sodhaganga. Studies focused on sleep quality, disturbances, and related factors among individuals with KHOA were screened. Finally, 17 articles were included in the final analysis.

**Results:**

Depression and elevated pain levels emerged as prominent contributors to sleep disturbances in individuals with KHOA. The meta-analysis revealed a pooled effect size of 8.53 (95% CI: 7.18–9.87) in Pittsburgh Sleep Quality Index (PSQI) scores, indicating significantly poorer sleep quality among patients with knee and/or hip osteoarthritis compared to healthy controls (*p* < 0.0001). However, there was substantial heterogeneity across studies (*I*^2^ = 94.96%).

**Conclusion:**

This study highlights that individuals with knee and/or hip osteoarthritis experience significantly impaired sleep quality, primarily driven by pain and psychological factors. The findings underscore the need for integrated clinical approaches that address not only the physical symptoms of OA but also its broader impact on sleep and mental health.

**Systematic review registration:**

[https://www.crd.york.ac.uk/prospero/], identifier [CRD42024547589].

## 1 Introduction

Osteoarthritis (OA) contributes to persistent pain and reduced mobility, particularly affecting weight-bearing joints, such as the knees and hips (1–3). It is characterized by cartilage degeneration, leading to joint stiffness, pain, and functional limitations (1). Disrupted sleep is a frequently reported comorbidity in individuals with OA, significantly impacting their quality of life (3, 4) by altering the body’s homeostasis (5). Sleep disturbances are directly proportional to worse pain outcomes (6) and act as a catalyst for pain induction and the creation of new illnesses, such as memory deficit and cognitive impairments, etc., (7). Studies suggest a reciprocal relationship between sleep and OA, where pain can disrupt sleep, and poor sleep quality can exacerbate pain perception (8). The current factsheet evidences a 113% increase in OA cases from 1990 to 2019, accounting for about 528 million cases globally, of which 73% of the population belongs to the more than 55 years age group (9), with 365 million knee OA sufferers, followed by hip (10, 11). There is a future prediction of a rise in OA until 2050, which will increase the global health burden (12). OA, along with sleep disturbances, results in a double health burden with functional limitations, psychosocial dysfunction, and increased healthcare utilization, imposing significant economic cost to society (13, 14).

Understanding the relationship between sleep disturbances/quality and knee and/or hip osteoarthritis (KHOA) in individuals with KHOA is crucial for developing effective management strategies. There is a lack of evidence regarding sleep assessment in knee and hip OA till now (15). Secondly, most studies merged different classifications of arthritis pain into a unified category against personalized effects on sleep. Thirdly, there is a lack of current publications focusing on specific joint affections instead of symptomatic OA (16). To date, there is no systematic review on the prevalence and assessment of sleep disturbances and sleep quality in individuals diagnosed with knee and hip OA, which is a matter of concern carrying the utmost significance. This systematic review assesses the existing scientific literature on this topic comprehensively and offers crucial insights to inform clinical practice and guide future research efforts in improving sleep for individuals with KHOA.

### 1.1 Objectives

- To estimate the extent of sleep disturbances in individuals with KHOA.
- To identify factors influencing sleep disturbances in individuals KHOA.
- To assess various aspects of sleep quality, including type, pattern, duration, onset time, and efficiency.

## 2 Materials and methods

Adhering to the Preferred Reporting Items for Systematic Reviews and Meta-Analyses (PRISMA) guidelines (17, 18), this systematic review and meta-analysis were performed to evaluate and consolidate information on sleep issues and quality in KHOA (PRISMA 2020 checklist in Supplementary Table 1). Before completing the main search, the protocol was documented and registered in the PROSPERO database under the registration number CRD42024547589.

### 2.1 Study selection criteria

Inclusion criteria:

- Studies investigating sleep disturbances or various aspects of sleep quality in patients with KHOA
- Availability of full-text articles
- Articles published in English within the past 20 years (2004–2024)

Exclusion criteria:

- Case reports, case series, reviews (including literature, scoping, systematic, and umbrella reviews), editorials, conference proceedings, policy briefs, letters, reports, synopses, and policy or program documents that do not provide evidence-based information on sleep issues and quality in KHOA patients
- Studies focusing on osteoarthritis in joints other than the knee or hip
- Studies that do not evaluate sleep disturbances, sleep quality, or interventions aimed at improving sleep in KHOA patients

### 2.2 Eligibility criteria

Population:

- Individuals of all age groups
- Clinically diagnosed KHOA as confirmed by a physician
- Self-reported KHOA patients with validated radiological or computed tomography (CT) scan findings

Exposure:

- Contributors to sleep disturbances such as pain, medication usage, psychological factors, and physical activity levels
- Strategies and interventions for improving sleep quality in individuals with KHOA

Outcomes:

- Prevalence of sleep disturbances concerning KHOA as primary outcomes
- Factors associated with sleep issues in individuals with KHOA
- Variations in sleep quality, including sleep onset, duration, efficiency, latency, types, and patterns

### 2.3 Search strategy

A systematic search was performed across electronic databases, including Medline via PubMed, CINAHL via EBSCO, EMBASE, Scopus, ScienceDirect, and Web of Science. The structure of the search strategy was prepared in accordance with the research question’s requirements and the specific formats required by the databases. Additionally, gray literature was explored using Google Scholar, ProQuest, and Sodhganga.

“Sleep disorders,” “sleep deprivation,” “sleep disturbance,” “sleep variation,” “sleep quality,” “sleep quantity,” “sleep wake disorders,” “circadian rhythm,” “sleep disorders, circadian rhythm,” “osteoarthritis,” “knee osteoarthritis,” and “hip osteoarthritis” have been applied as search terms. The comprehensive search strategy for the above databases is available in Supplementary Table 1.

### 2.4 Study screening and selection

Once the data and references were collected, they were converted into multiple formats, such as CSV and RIS. The studies were then imported into the Rayyan QCRI software to identify and remove duplicates. The screening process was performed in two stages: first, an initial title and abstract screening, followed by a full-text review.

During the title-abstract screening, three independent reviewers evaluated the studies based on predefined inclusion and exclusion criteria. Any conflicts were resolved through discussion with a third reviewer. Full-text articles that satisfy the inclusion criteria were then thoroughly assessed by two impartial reviewers. Disagreements arose regarding the eligibility of certain studies, prompting a third consulting reviewer to intervene and facilitate a resolution.

A record of excluded articles from the full-text screening was maintained, along with documented reasons for exclusion based on eligibility criteria. Relevant studies were categorized according to study design, osteoarthritis, and sleep components, and then systematically organized into a tabulated excel sheet.

### 2.5 Data extraction

**FIGURE 1:**
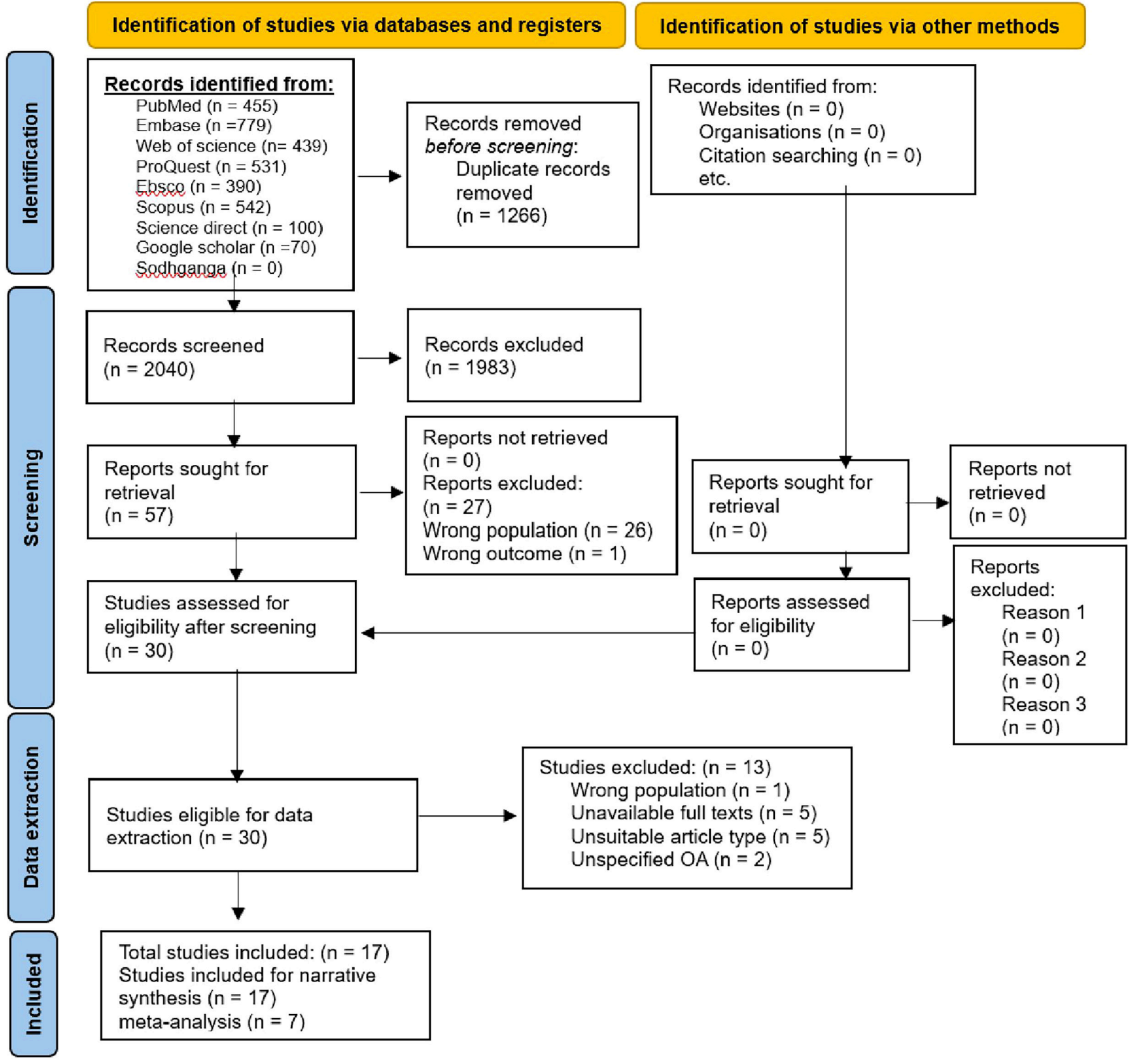
Flow chart for systematic review process.

A pre-prepared pilot standard coding form containing the study type, study setting, study period, sample size, publication year, authors, osteoarthritis assessment criteria/tool, and sleep-related judgment criteria, was utilized for significant data extraction from included studies for evidence synthesis concerning the impact of KHOA on sleep including the overall objective. The literature screening process and results are provided through the PRISMA flowchart (Figure 1).

The data extraction sheet included study characteristics, participant characteristics, KHOA assessment tools, sleep assessment methods, outcomes related to sleep disturbances and sleep quality, main findings and conclusions.

### 2.6 Study of quality appraisal to assess risk of bias

Quality appraisal was carried out independently by two reviewers. The risk of bias was assessed in accordance with the Joanna Briggs Institute (JBI) guidelines. (19)

### 2.7 Statistical method

A narrative synthesis was conducted in accordance with the Synthesis Without Meta-Analysis (SWiM) guidelines (20) to examine sleep disturbances related to KHOA. Data have been analyzed using RStudio (version 2024 12.0 + 467) software. Both non-continuous and continuous variables were reported with 95% confidence intervals (CIs), and forest plots were generated. Heterogeneity across studies was evaluated using I^2^ statistics and the *Q*-test. When the *P*-value was ≥ 0.10 and *I*^2^ < 50%, indicating homogeneity, a fixed-effects model was applied. Conversely, when the *P*-value was < 0.10 and *I*^2^ ≥ 50%, suggesting significant heterogeneity, a random-effects model was utilized. A *P*-value less than 0.05 is considered statistically significant. A sensitivity analysis was conducted to determine the extent of variation in the pooled effect size across various studies by systematically removing individual studies. Subgroup analysis could not be performed due to limited data for comparisons and the presence of extreme heterogeneity.

### 2.8 Risk of bias assessment

After applying the JBI critical appraisal tool, studies were classified as low quality if they achieved a score below 50%, moderate quality if they achieved a score between 50% and 70%, and high quality if they scored above 70%.

## 3 Results

A total of 3,306 relevant articles were retrieved. Following deduplication, 2,040 articles were screened. After the title-abstract review, 30 articles met the inclusion criteria. Following a full-text review, 13 articles were excluded, including five due to unavailability of full texts, five with unsuitable article types, and three that did not specify the type of osteoarthritis or focused on conditions other than knee or hip osteoarthritis. Ultimately, 17 articles were included in this study.

### 3.1 Study characteristics

The study characteristics demonstrated that all articles fell within the medium to high quality range. The included studies consisted of one case-control study, two cohort studies, and 14 cross-sectional studies. The studies covered cases from Brazil, Spain, China, the United Kingdom, Japan, Australia, Nigeria, and Canada (each with one study), South Korea (three studies), the United States, and Turkey (each with three studies).

Knee and/or hip osteoarthritis diagnosis and severity were determined using various criteria, including the National Institute for Health and Care Excellence (NICE) guidelines (21), American College of Rheumatology classification (22–26), International Classification of Diseases- Tenth revision (ICD-10) codes (27), and the Western Ontario and McMaster Osteoarthritis Index (WOMAC) questionnaire (16, 22–24, 28–30), which assesses pain, stiffness, and physical function. Additional measures included the Numerical Pain Rating Scale (NPRS, 0–10) for pain intensity (24, 25, 30, 31), Kellgren/Lawrence (K/L) grading for severity (15, 22, 23, 25, 29, 31–34), Knee Injury and Osteoarthritis Outcome Score (KOOS) (32), Lequesne Index for knee OA severity, Visual Analog Scale (VAS) (22, 26) for knee OA pain intensity, Hip Outcome Score (HOS) (16), modified Harris Hip Score (mHHS) (16), Steinbrocker functional classification, Hip Injury and Osteoarthritis Outcome Score (HOOS) questionnaire (25), and radiographic assessments.

The Pittsburgh Sleep Quality Index (PSQI) is a widely accepted and frequently used questionnaire for assessing sleep quality, completed subjectively by patients. A higher PSQI score indicates poorer sleep quality. Sleep disturbance assessment relied on the PSQI questionnaire in nine studies (16, 21–26, 30, 32), the Epworth Sleepiness Scale (ESS) in one study (21), the National Sleep Foundation’s sleep duration criteria in one study (31), the Medical Outcomes Study sleep questionnaire in one study (15), the ICD-10 disease code in one study (27), other self-assessment questionnaires in two studies (33, 34), and the Center for Epidemiologic Studies Depression Scale (CES-D) in two studies (28, 29). One study did not specify the sleep assessment tool used. The PSQI provides a global sleep score in addition to six sub-scores assessing sleep disturbance, sleep latency, sleep duration, subjective sleep quality, habitual sleep efficiency, use of sleep medications, and daytime somnolence, with individual domain scores from 0 to 3. Higher scores indicate poorer performance in that category.

Osteoarthritis diagnosis and sleep assessment tools were not specified clearly in one study by Feehan et al. (35).

Seven studies reported sleep disturbances or poor sleep quality among KHOA patients using various measures. The occurrence of sleep disturbances is higher in individuals with OA. All studies found a significant association between sleep quality and KHOA, indicating that greater KHOA severity is linked to poorer sleep quality. Four studies explicitly stated in their results that more than 55% of KHOA patients had a PSQI score of ≥ 5. Six studies identified additional influencing factors, including anxiety, depression, high pain scores, greater KHOA severity, and comorbidities. Regarding sleep disorders, one study reported that 20% of patients had obstructive sleep apnea (OSA), while another study found that 71% experienced a recent sleep problem, 56% reported having insomnia, and 56% reported inadequate sleep.

### 3.2 Quality appraisal to assess the risk of bias

The assessment was conducted using the critical appraisal tools from the JBI (19), as required (Supplementary Table 2). The quality assessment of the included articles revealed that cross-sectional studies received scores ranging from 5 to 8 out of 8, indicating moderate to high quality. Cohort studies received scores ranging from 8 to 11 out of 11, reflecting high-quality research. Similarly, case-control studies attained a perfect score of 10 out of 10, confirming their high quality. Table 1 represents the characteristics of the included studies of this systematic review.

**TABLE 1:** Characteristics of the included studies (*n* = 17).

Sl. no.	References	Type of osteoarthritis	Place of study	Study design	Sample size	Osteoarthritis severity assessment	Sleep parameters	Sleep disturbance assessment tools
1	Kakazu et al. (21)	Knee osteoarthritis	Brazil	Cross-sectional	451	Assessed based on the National Institute for Health and Care Excellence (NICE) guidelines	Sleep quality, excessive daytime sleepiness	PSQI and ESS
2	Garcia et al. (24)	Knee osteoarthritis	Spain	Cross-sectional	36	• Diagnosed according to American College of Rheumatology classification • WOMAC questionnaire, which includes dimensions of pain, stiffness, and physical function • For pain intensity - numerical pain rate scale (NPRS, 0–10)	Sleep efficiency, duration, latency, onset, total sleep time	PSQI
3	Jacob et al. (27)	Various types including polyosteoarthritis, hip osteoarthritis, knee osteoarthritis, and unspecified osteoarthritis	UK	Case-control	351,932 adults	Diagnosed using ICD-10 codes	Sleep efficiency, duration, latency, total sleep time	ICD-10: F51
4	Cho et al. (31)	Knee osteoarthritis	South Korea	Cross-sectional	8,918 adults	• Assessed using the Kellgren-Lawrence (KL) grading scale • For pain- numeric rating scale (NRS) • Radiological diagnosis via X-ray	Duration, with categories of short (< 6 h), normal (7–8 h), and long (≥ 9 h)	National Sleep Foundation’s sleep time duration criteria recommendations
5	Wang et al. (28)	Knee osteoarthritis	China	Observational (cohort)	3,813 participants	Assessed using the Western Ontario and McMaster Osteoarthritis Index (WOMAC) pain scale	Sleep quality, sleep disturbance frequency	Center for Epidemiologic Studies Depression Scale (CES-D)
6	Tubanur Yilmaz et al. (23)	Knee osteoarthritis	Turkey	Cross-sectional study	151 KOA patients	• American College of Rheumatol ogy (ACR) • Western Ontario and McMaster Universities Osteoarthritis Index (WOMAC) • Kellgren-Lawrence Radiological Staging in Osteoarthritis (KLRSO)	1) Sleep quality 2) Sleep latency 3) Sleep duration 4) Sleep efficiency 5) Daytime sleep dysfunction 6) Sleep disturbance	PSQI
7	Sasaki et al. (32)	Knee osteoarthritis	Japan	Cohort study	1,214 participants (456 men and 758 women)	• Severity by Kellgren/Lawrence (K/L) grade, and joint space widths were measured • Presence of nocturnal knee pain and Knee Injury and Osteoarthritis Outcome Scores (KOOS) were assessed • Self-completed questionnaires	1) Sleep quality 2) Sleep latency 3) Sleep duration 4) Habitual sleep efficiency 5) Sleep disturbance 6) Use of sleeping medication 7) Daytime sleep dysfunction	PSQI
8	Allen et al. (15)	Knee/hip osteoarthritis	USA	Cross-sectional study	Total = 2,682 (first follow up = 1,687 + newly enrolled group = 995)	• Posteroanterior radiography of both knees with weight-bearing, using the SynaFlex^®^ positioning frame • Kellgren-Lawrence (K-L) score	1) Trouble falling asleep 2) Trouble staying asleep 3) Waking early 4) Daytime sleepiness 5) Not enough sleep 6) Not feeling rested	Medical outcomes study sleep questionnaire
9	Afşar et al. (22)	Knee osteoarthritis	Turkey	Cross sectional study	41 (27 female,14 male)	• American College of Rheumatology (ACR) • Kellgren-Lawrence (KL) grading scale • Visual Analog Scale (VAS)- pain intensity of knee OA • Western Ontario and McMaster Universities Osteoarthritis Index (WOMAC)- intensity of pain, stiffness, and level of function due to knee OA • Lequesne Index- severity of knee OA	1) Sleep disturbances 2) Sleep latency 3) Daytime dysfunction 4) Habitual sleep efficiency 5) Subjective sleep quality 6) Use of sleeping medication	PSQI
10	Park et al. (33)	Knee/hip osteoarthritis	South Korea	Cross sectional study [Fifth Korea National Health and Nutrition Examination Survey (KNHANES-V)]	5,268	Kellgren–Lawrence grading system	Sleep duration (5, 6, 7–8, and 9 h)	Self-reported questionnaire
11	Fu et al. (25)	Hhip osteoarthritis	Australia	Cohort study (Internet-based case-crossover study)	252	• American College of Rheumatology criteria • Kellgren-Lawrence grade • Numeric rating scale (NRS) • Hip injury and Osteoarthritis Outcome Score (HOOS) questionnaire	1) Sleep quality 2) Sleep duration 3) Sleep disturbance 4) Sleep latency 5) Daytime dysfunction 6) Sleep efficiency 7) Sleep medication	PSQI
12	Kiyak et al. (26)	Knee osteoarthritis	Turkey	Descriptive study	90	Pain- visual analog scale (VAS)	Poor sleep quality	PSQI
13	Martinez et al. (16)	Hip osteoarthritis	USA	Prospective, cross-sectional study	106	• Western Ontario and McMaster Universities Osteoarthritis Index (WOMAC) • Hip outcome score (HOS) • Modified Harris hip score (mHHS) • Visual analogue pain scale (EQ-VAS)	1) Sleep quality 2) Sleep latency 3) Sleep duration 4) Habitual sleep efficiency 5) Sleep disturbance 6) Use of sleep medication 7) Day time dysfunction	PSQI
14	Jung et al. (34)	Knee/hip osteoarthritis	South Korea	Cross-sectional observational study	11,540 participants (4,915 men and 6,625 women)	• Self-reported questionnaire • Knee/hip joint radiographs • Kellgren-Lawrence (KL) grade	Sleep duration (0–3, 4–5, 6–7, and ≥ 8 h)	Self-report questionnaire
15	Lapane et al. (29)	Knee osteoarthritis	USA	Cross-sectional and longitudinal study analysis of osteoarthritis Initiative (OAI) data	1) 2,517 participants for the baseline cross-sectional analyses 2) 1,882 participants for the longitudinal analyses	• Radiographs • Western Ontario and McMaster Universities Arthritis Index (WOMAC) • Five-point Likert scale • Kellgren Lawrence grade (K-L)	Restless sleep (< 1 night, 1–2 nights, 3–4 nights, 5–7 nights)	Center for Epidemiologic Studies Depression Scale (CES-D)
16	Akintayo et al. (30)	Knee osteoarthritis	Nigeria	Multi-center, hospital-based, cross-sectional study	250 (50 patients per center)	• Pain severit- numerical rating scale (NRS) • Western Ontario and McMaster Universities Arthritis Index (WOMAC) • Functional classification- Steinbrocker functional classification • Radiographs • Kellgren and Lawrence (KL) criteria • Five-point Likert scale	Sleep quality (scores less than 5 = no sleep ab normality and score ≥ 5 = poor sleep quality)	PSQI
17	Feehan et al. (35)	Knee osteoarthritis	Canada	Cross-sectional cohort study	172 (30% knee OA)	Not specified	High and low sleepers: > 81/2 and < 61/2 h sleep, respectively	Not specified

### 3.3 Meta-analysis

**FIGURE 2:**
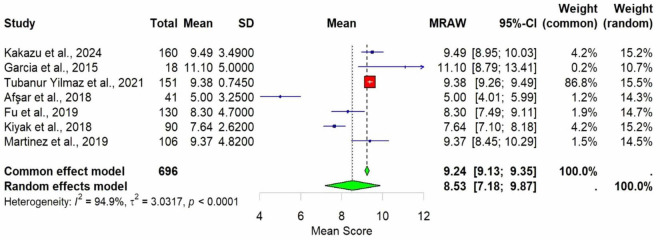
Forest plot of poor sleep among KHOA^a^ patients based on PSQI^b^ scores. Mean is used as an effect size.

**FIGURE 3:**
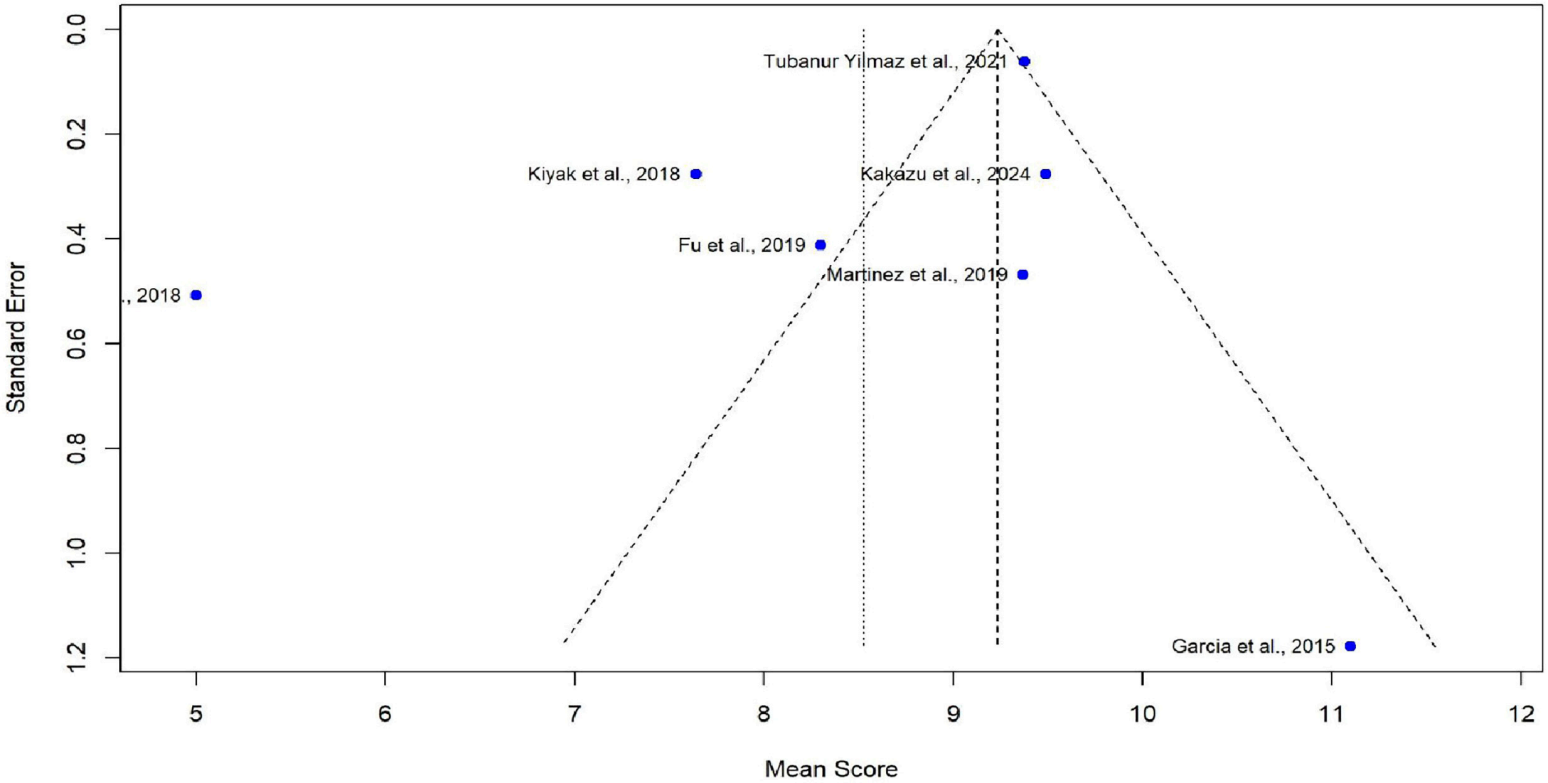
Funnel plot of poor sleep among KHOA^a^ patients based on PSQI^b^ scores.

**FIGURE 4:**
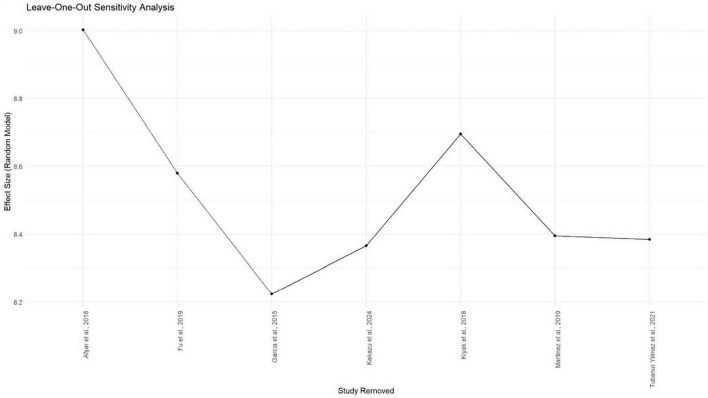
Gg plot depicting the impact of individual studies on the PSQI^a^ mean score effect size through the random effect model.

Meta-analysis was conducted using seven studies that employed a common sleep assessment tool, with PSQI score as the outcome measure for KHOA patients, reported in terms of mean and standard deviation (Figures 2–4). The pooled mean PSQI score among individuals with KHOA was 8.53, which is above the standard cutoff of 5, indicating poor sleep quality. In other words, the average PSQI score among KHOA patients was 8.53, which exceeds the established threshold of 5 for poor sleep quality. So, we can be 95% confident that the true effect size lies within the interval 7.18–9.87, indicating a statistically significant overall effect. The relatively narrow confidence interval suggests a precise estimate with a low likelihood of results occurring by chance. Heterogeneity analysis revealed substantial variability across studies, with an I^2^ of 94.96%, indicating extremely high heterogeneity. A *p*-value < 0.0001 and *H*^2^ = 39.31 further confirm considerable heterogeneity. The estimated level of variance (τ^2^ = 3.0317) across studies justified the application of the random-effects model in the meta-analysis. The forest plot showed that Afsar et al. (22) had the lowest mean score (5.00), while Alburquerque-García et al. (24) had the highest (11.10). The funnel plot indicated an outlier in the lower-right region Alburquerque-García et al. (24), suggesting potential true heterogeneity due to variations in study populations. A leave-one-out sensitivity analysis was performed to evaluate the robustness of a meta-analysis systematically and to identify whether a single study had a disproportionate influence on the overall results. Ggplot showed that removing Afşar et al. (22), Alburquerque-García et al. (24) significantly impacted the effect size, increasing it to approximately 9.0 or decreasing it to around 8.2, respectively, highlighting heterogeneity in the overall estimate. This high heterogeneity (*I*^2^ = 94.96%) makes interpretation of the pooled estimate more challenging. Several factors may explain this variability. First, differences in study populations could have contributed; some studies included younger patients, while others had older or more severe cases, which can affect sleep patterns. Second, cultural and regional differences in sleep habits and healthcare access may have influenced the reporting of PSQI. Third, although all studies used PSQI, the application of the tool may have varied (for example, in cut-off thresholds, versions used, or interviewer versus self-administered assessments). Fourth, differences in study design and sample size may also contribute to variability. Finally, co-morbidities such as depression, obesity, or medication use were inconsistently reported across studies and might explain variations in sleep quality.

Even though subgroup analysis was not feasible due to the small number of studies, a narrative interpretation suggests that both clinical and methodological diversity are likely sources of heterogeneity. This indicates that while the pooled effect supports an overall trend of poor sleep in KHOA patients, caution is warranted in generalizing the exact magnitude of the effect. These findings underscore the need for future research with more standardized study designs and better control for confounding variables, which will be critical to refining our understanding of sleep problems in KHOA and guiding targeted clinical interventions.

## 4 Discussion

As previously defined, this study aims to provide a comprehensive understanding of the prevalence and characteristics of sleep disturbances and sleep quality in KHOA individuals, as well as the impact of KHOA on sleep patterns. It also examines the contributing factors and sleep assessment tools used in these evaluations. The findings indicate that KHOA individuals have significantly poorer sleep quality and a higher risk of sleep disturbances compared to the healthy population. Based on PSQI scores, they demonstrate lower subjective sleep quality, longer sleep latency, shorter sleep duration, and a higher prevalence of sleep disorders. Nevertheless, no significant differences were observed in the use of sleep medications, sleep efficiency, daytime dysfunction, or reliance on sleeping aids. Additionally, KHOA patients have a higher likelihood of experiencing unhealthy sleep durations, including both insufficient and excessive sleep, compared to healthy individuals.

Our systematic review confirms previous findings that patients with OA have a higher likelihood of experiencing sleep disturbances (36, 37). Recognizing the gap in sleep data within knee OA trials, the authors stressed the need for formal sleep assessments to evaluate their impact on treatment outcomes, leading to this systematic review (8). Evidence suggests that individuals with KHOA are prone to daytime lethargy and sleepiness, largely attributed to poor sleep quality, nocturnal pain, and other contributing factors (16, 38). However, we found no considerable difference in daytime dysfunction between KHOA individuals and the healthy population.

Findings from the PSQI in these studies revealed considerable variability in total sleep duration among KHOA patients, with a pronounced tendency toward insufficient sleep rather than excessive sleep (16, 25).

Pain, inflammation, depression, and anxiety are frequently linked to short sleep duration and reduced sleep quality in KHOA patients (39). Research revealed that targeted interventions, including cognitive-behavioral therapy, effective pain management, and mind-body practices like yoga, can help alleviate sleep disturbances in OA patients by addressing these key contributing factors (40–42).

Martinez et al. (16) reported that 20% of patients with symptomatic hip osteoarthritis had a history of OSA, highlighting a clinically significant overlap likely due to shared risk factors and effects on pain and function (16). Evidence from previous studies suggests that OSA is considerably more common among OA patients than among the general population, occurring in 66% of OA patients compared to 17% of the general population (43, 44). Both conditions are strongly associated with obesity, aging, and systemic inflammation, likely contributing to their co-occurrence. Hip OA pain disrupts sleep architecture, resulting in delayed sleep onset, reduced sleep efficiency, and daytime fatigue, while poor sleep further amplifies pain sensitivity, creating a self-perpetuating cycle. OSA-induced intermittent hypoxia elevates inflammatory mediators, including IL-6 and TNF-alpha, which sensitize nociceptive pathways and exacerbate functional impairment such as reduced physical activity, increased fatigue, and poorer WOMAC scores, an independent predictor of poor sleep. Screening for OSA enables targeted interventions, such as continuous positive airway pressure (CPAP) therapy, which can improve sleep, alleviate pain, and enhance postoperative recovery, underscoring the value of a holistic, multidisciplinary approach to OA management (45).

Furthermore, a bidirectional relationship exists between KHOA and sleep disturbances, where poor sleep can result in the onset or worsening of KHOA symptoms (29). This cyclical interaction is influenced by pain, inflammation, psychological distress, and neurobiological factors. KHOA-related joint pain often reduces physical activity, further disrupting sleep patterns. Nocturnal pain, inflammation, psychological distress, and coexisting sleep disorders contribute to fragmented and poor-quality sleep. Inadequate sleep increases pain sensitivity, promotes inflammation, accelerates cartilage degeneration, and disrupts metabolism, ultimately driving OA progression. As OA symptoms further disrupt sleep, the resulting sleep disturbances, in turn, exacerbate OA, creating a self-perpetuating cycle of disease worsening.

In a few of the included studies, BMI was either not reported or, when available (27), its potential impact on sleep quality was not assessed. Although obesity was occasionally identified as a comorbidity, it was generally mentioned only superficially and not examined in relation to BMI as a contributing factor to impaired sleep. Furthermore, in OA, excessive mechanical loading and activity-induced synovitis during daily activities contribute to knee pain, which in turn adversely affects sleep quality.

### 4.1 Strength and limitations

This is the inaugural systematic review focused on mapping the existing literature and assessing sleep appraisal in KHOA, a critical and significant area of concern, by analyzing data from 17 studies. Our review confirms a significant positive correlation between sleep disturbances/quality and osteoarthritis concerning KHOA. The first limitation is that only studies focusing on knee and/or hip OA are included to assess their impact on sleep quality, while other types of osteoarthritis are excluded. Additionally, the search is restricted to studies published within the last 20 years, potentially omitting relevant findings from earlier years. Furthermore, limiting the analysis to English-language studies may exclude valuable insights available in other languages. Further research should explore sleep-specific interventions for individuals with KHOA and their potential effect on disease activity and overall quality of life. Many of the studies used self-reported questionnaires, such as the PSQI, ESS, and CES-D, with very few employing objective measures like polysomnography or other formal sleep studies. This is particularly important because self-reported data are inherently subject to bias and may not accurately reflect actual sleep patterns or disturbances.

## 5 Conclusion and recommendation

This review evidences the current status of sleep, addressing the gaps in sleep assessment among KHOA individuals, thereby finding a better way to combat the bifold struggle with sleep dysfunction and most popular, degenerative joint disorder OA, knee and hip specific to people in declining years. Pain, inflammation, comorbidities, depression, and anxiety were contributing factors for substandard sleep in KHOA individuals. Our meta-analysis confirms the significant association between lower sleep quality and KHOA individuals compared to the healthy population. This included poor sleep quality, longer sleep latency, and a higher risk of unhealthy sleep duration, such as insufficient sleep or excessive sleep. This underscores the need for targeted interventions to manage pain and mental health in order to improve sleep outcomes. Therefore, national and clinical guidelines for osteoarthritis management should incorporate routine screening for sleep disturbances, including assessments of insomnia and sleep quality, as a standard component of care. Globally, sleep health should be systematically integrated into chronic disease care models through ongoing national health programs to enhance patient outcomes, improve quality of life, and ensure more comprehensive, person-centered care. In the future, additional large-scale prospective studies are essential to further support patient management and treatment.

## Data Availability

The original contributions presented in this study are included in this article/Supplementary material, further inquiries can be directed to the corresponding author.
