# Supplementary material for: Sleep disturbances and sleep quality among individuals diagnosed with osteoarthritis: a systematic review and meta-analysis
Source: Front Med (Lausanne). 2025 Nov 19;12:1653047. doi: 10.3389/fmed.2025.1653047 (PMC12672537; doi:10.3389/fmed.2025.1653047)
Supplement: Supplementary file 1 [file Table_1.docx]

**Supplementary Table 1: Search strategy of one database with search terms**

| Search strategy 1– Medline via Pubmed | | |
| --- | --- | --- |
| Search strategy | Query | Result |
| 1. | **"Sleep Initiation and Maintenance Disorders"[Mesh] OR "Sleep Wake Disorders"[Mesh] OR "Dyssomnias" [Mesh]) OR (Intrinsic Sleep Disorder*[Title/Abstract] OR Sleep Disorder, Intrinsic*[Title/Abstract] OR Sleep State Misperception*[Title/Abstract] OR Hypersomnia*, Post-Traumatic[Title/Abstract] OR Hypersomnia, Post Traumatic[Title/Abstract] OR Hypersomnias, Post-Traumatic Post-Traumatic Hypersomnia*[Title/Abstract] OR Hypersomnia*, Posttraumatic[Title/Abstract] OR Posttraumatic Hypersomnia*[Title/Abstract] OR Disorders of Initiating[Title/Abstract] AND Maintaining Sleep[Title/Abstract] OR Sleeplessness[Title/Abstract] OR Insomnia Disorder*[Title/Abstract] OR Insomnia*[Title/Abstract] OR Chronic Insomnia[Title/Abstract] OR Insomnia, Chronic[Title/Abstract] OR Early Awakening[Title/Abstract] OR Awakening, Early[Title/Abstract] OR Nonorganic Insomnia[Title/Abstract] OR Insomnia, Nonorganic[Title/Abstract] OR Primary Insomnia[Title/Abstract] OR Insomnia, Primary[Title/Abstract] OR Psychophysiological Insomnia[Title/Abstract] OR Insomnia, Psychophysiological[Title/Abstract] OR Rebound Insomnia[Title/Abstract] OR Insomnia, Rebound[Title/Abstract] OR Secondary Insomnia[Title/Abstract] OR Insomnia, Secondary[Title/Abstract] OR Sleep Initiation Dysfunction[Title/Abstract] OR Dysfunction*, Sleep Initiation[Title/Abstract] OR Sleep Initiation Dysfunctions[Title/Abstract] OR Transient Insomnia[Title/Abstract] OR Insomnia, Transient[Title/Abstract] OR Parasomnia[Title/Abstract] OR Sensory Paroxysm*, Sleep[Title/Abstract] OR Paroxysm*, Sleep Sensory[Title/Abstract] OR Sleep Sensory Paroxysm*[Title/Abstract] OR Deprivation, Sleep[Title/Abstract] OR Insufficient Sleep Syndrome*[Title/Abstract] OR Syndrome, Insufficient Sleep[Title/Abstract] OR REM Sleep Deprivation[Title/Abstract] OR Deprivation, REM Sleep[Title/Abstract] OR Sleep Deprivation, REM[Title/Abstract] OR Sleep Fragmentation[Title/Abstract] OR Fragmentation, Sleep[Title/Abstract] OR Insufficient Sleep[Title/Abstract] OR Sleep, Insufficient[Title/Abstract] OR Inadequate Sleep[Title/Abstract] OR Sleep, Inadequate[Title/Abstract] OR Sleep Debt[Title/Abstract] OR Sleep Insufficiency*[Title/Abstract] OR Insufficiency*, Sleep[Title/Abstract] OR Disorder*, Sleep Wake[Title/Abstract] OR Sleep Wake Disorder[Title/Abstract] OR Wake Disorder*, Sleep[Title/Abstract] OR Sleep Disorder*[Title/Abstract] OR Disorder*, Sleep[Title/Abstract] OR Long Sleeper Syndrome*[Title/Abstract] OR Sleeper Syndrome*, Long[Title/Abstract] OR Syndrome*, Long Sleeper[Title/Abstract] OR Short Sleeper Syndrome*[Title/Abstract] OR Sleeper Syndrome*, Short[Title/Abstract] OR Syndrome*, Short Sleeper[Title/Abstract] OR Short Sleep Phenotype*[Title/Abstract] OR Phenotype*, Short Sleep[Title/Abstract] OR Sleep Phenotypes, Short[Title/Abstract] OR Dyssomnia[Title/Abstract] OR Sleep Disorder*, Extrinsic[Title/Abstract] OR Extrinsic Sleep Disorder*[Title/Abstract] OR Adjustment Sleep Disorder*[Title/Abstract] OR Sleep Disorder*, Adjustment[Title/Abstract] OR Sleep Disturbance*[Title/Abstract] OR Somnipathy[Title/Abstract] OR Sleep Problem*[Title/Abstract] OR Sleep Issue*[Title/Abstract] OR Sleep Disruption[Title/Abstract] OR Sleep Dysfunction[Title/Abstract] OR Sleep Difficult*[Title/Abstract] OR Sleep Deprivation[Title/Abstract] OR Restless Sleep[Title/Abstract] OR Sleep restless*[Title/Abstract] OR Delayed Sleep Phase Syndrome[Title/Abstract] OR Delayed Sleep-Phase Syndrome*[Title/Abstract] OR Non-24 Hour Sleep-Wake Disorder[Title/Abstract] OR Non 24 Hour Sleep Wake Disorder[Title/Abstract] OR Sleep-Wake Disorder, Non-24 Hour[Title/Abstract] OR Sleep Wake Disorder, Non 24 Hour[Title/Abstract] OR Nonorganic Sleep Wake Cycle Disorder*[Title/Abstract])** Filters: **from 2004 - 2024** | 123954 |
| 2. | **("Sleep Quality"[Mesh] OR "Sleep Hygiene"[Mesh]) OR (Sleep qualit*[Title/Abstract] OR Qualit*, Sleep[Title/Abstract] OR Sleeping Habit*[Title/Abstract] OR Sleep Habit*[Title/Abstract] OR Habit*, Sleep[Title/Abstract] OR Habit*, Sleep*[Title/Abstract] OR Hygiene, Sleep[Title/Abstract] OR Good Sleep Habit*[Title/Abstract] OR Habit*, Good Sleep[Title/Abstract] OR Sleep Habit*, Good[Title/Abstract] OR Duration, Sleep[Title/Abstract] OR Sleep Quantity*[Title/Abstract] OR Quantity, Sleep[Title/Abstract] OR Total Sleep Time[Title/Abstract] OR Latency, Sleep[Title/Abstract] OR Sleep Latenc*[Title/Abstract] OR Sleep Onset Latenc*[Title/Abstract] OR Latency, Sleep Onset[Title/Abstract] OR Onset Latencies, Sleep[Title/Abstract] OR Sleep Pattern[Title/Abstract] OR Sleep Depth[Title/Abstract] OR Sleep Consistency[Title/Abstract] OR Sleep Efficiency[Title/Abstract] OR Sleep Satisfaction[Title/Abstract] OR Sleep Architecture[Title/Abstract] OR Restfulness[Title/Abstract] OR Sleep Soundness[Title/Abstract] OR Sleep Wellness[Title/Abstract] OR Sleep Health[Title/Abstract] OR Sleep State[Title/Abstract] OR Sleep Status[Title/Abstract] OR Sleep Continuity[Title/Abstract] OR Sleep Regularity[Title/Abstract] OR Sleep Variabilit*[Title/Abstract] OR Sleep Maintenance[Title/Abstract] OR Sleep Hygiene[Title/Abstract] OR Sleep fragmentation[Title/Abstract])** Filters: **from 2004 - 2024** | 43461 |
| 3. | **("Osteoarthritis"[Mesh] OR "Osteoarthritis, Knee"[Mesh] OR "Osteoarthritis, Hip"[Mesh]) OR (Osteoarthritides[Title/Abstract] OR Arthriti*, Degenerative[Title/Abstract] OR Degenerative Arthriti*[Title/Abstract] OR Osteoarthros*[Title/Abstract] OR Osteoarthrosis Deformans[Title/Abstract] OR Arthros*[Title/Abstract] OR Knee Osteoarthriti*[Title/Abstract] OR Osteoarthritis of the Knee[Title/Abstract] OR Osteoarthritis of Knee[Title/Abstract] OR Hip Osteoarthritis[Title/Abstract] OR Osteoarthritis of the Hip[Title/Abstract] OR Coxarthros*[Title/Abstract] OR Osteoarthritis Of Hip*[Title/Abstract] OR Wear-and-tear Arthritis[Title/Abstract] OR Joint degeneration[Title/Abstract] OR Cartilage Degradation[Title/Abstract] OR Bone Spur*[Title/Abstract] OR Spur*, Bone[Title/Abstract] OR Osteophyt*[Title/Abstract] OR Joint Stiffness[Title/Abstract] OR Joint Inflammation[Title/Abstract] OR Joint Effusion[Title/Abstract] OR Hydrarthroses[Title/Abstract])** Filters: **from 2004 - 2024** | 108675 |
| 4. | **#1 OR #2**  **(("Sleep Quality"[Mesh] OR "Sleep Hygiene"[Mesh]) OR (Sleep qualit*[Title/Abstract] OR Qualit*, Sleep[Title/Abstract] OR Sleeping Habit*[Title/Abstract] OR Sleep Habit*[Title/Abstract] OR Habit*, Sleep[Title/Abstract] OR Habit*, Sleep*[Title/Abstract] OR Hygiene, Sleep[Title/Abstract] OR Good Sleep Habit*[Title/Abstract] OR Habit*, Good Sleep[Title/Abstract] OR Sleep Habit*, Good[Title/Abstract] OR Duration, Sleep[Title/Abstract] OR Sleep Quantity*[Title/Abstract] OR Quantity, Sleep[Title/Abstract] OR Total Sleep Time[Title/Abstract] OR Latency, Sleep[Title/Abstract] OR Sleep Latenc*[Title/Abstract] OR Sleep Onset Latenc*[Title/Abstract] OR Latency, Sleep Onset[Title/Abstract] OR Onset Latencies, Sleep[Title/Abstract] OR Sleep Pattern[Title/Abstract] OR Sleep Depth[Title/Abstract] OR Sleep Consistency[Title/Abstract] OR Sleep Efficiency[Title/Abstract] OR Sleep Satisfaction[Title/Abstract] OR Sleep Architecture[Title/Abstract] OR Restfulness[Title/Abstract] OR Sleep Soundness[Title/Abstract] OR Sleep Wellness[Title/Abstract] OR Sleep Health[Title/Abstract] OR Sleep State[Title/Abstract] OR Sleep Status[Title/Abstract] OR Sleep Continuity[Title/Abstract] OR Sleep Regularity[Title/Abstract] OR Sleep Variabilit*[Title/Abstract] OR Sleep Maintenance[Title/Abstract] OR Sleep Hygiene[Title/Abstract] OR Sleep fragmentation[Title/Abstract]) AND (2004:2024[pdat])) OR (("Sleep Initiation and Maintenance Disorders"[Mesh] OR "Sleep Wake Disorders"[Mesh] OR "Dyssomnias" [Mesh]) OR (Intrinsic Sleep Disorder*[Title/Abstract] OR Sleep Disorder, Intrinsic*[Title/Abstract] OR Sleep State Misperception*[Title/Abstract] OR Hypersomnia*, Post-Traumatic[Title/Abstract] OR Hypersomnia, Post Traumatic[Title/Abstract] OR Hypersomnias, Post-Traumatic Post-Traumatic Hypersomnia*[Title/Abstract] OR Hypersomnia*, Posttraumatic[Title/Abstract] OR Posttraumatic Hypersomnia*[Title/Abstract] OR Disorders of Initiating[Title/Abstract] AND Maintaining Sleep[Title/Abstract] OR Sleeplessness[Title/Abstract] OR Insomnia Disorder*[Title/Abstract] OR Insomnia*[Title/Abstract] OR Chronic Insomnia[Title/Abstract] OR Insomnia, Chronic[Title/Abstract] OR Early Awakening[Title/Abstract] OR Awakening, Early[Title/Abstract] OR Nonorganic Insomnia[Title/Abstract] OR Insomnia, Nonorganic[Title/Abstract] OR Primary Insomnia[Title/Abstract] OR Insomnia, Primary[Title/Abstract] OR Psychophysiological Insomnia[Title/Abstract] OR Insomnia, Psychophysiological[Title/Abstract] OR Rebound Insomnia[Title/Abstract] OR Insomnia, Rebound[Title/Abstract] OR Secondary Insomnia[Title/Abstract] OR Insomnia, Secondary[Title/Abstract] OR Sleep Initiation Dysfunction[Title/Abstract] OR Dysfunction*, Sleep Initiation[Title/Abstract] OR Sleep Initiation Dysfunctions[Title/Abstract] OR Transient Insomnia[Title/Abstract] OR Insomnia, Transient[Title/Abstract] OR Parasomnia[Title/Abstract] OR Sensory Paroxysm*, Sleep[Title/Abstract] OR Paroxysm*, Sleep Sensory[Title/Abstract] OR Sleep Sensory Paroxysm*[Title/Abstract] OR Deprivation, Sleep[Title/Abstract] OR Insufficient Sleep Syndrome*[Title/Abstract] OR Syndrome, Insufficient Sleep[Title/Abstract] OR REM Sleep Deprivation[Title/Abstract] OR Deprivation, REM Sleep[Title/Abstract] OR Sleep Deprivation, REM[Title/Abstract] OR Sleep Fragmentation[Title/Abstract] OR Fragmentation, Sleep[Title/Abstract] OR Insufficient Sleep[Title/Abstract] OR Sleep, Insufficient[Title/Abstract] OR Inadequate Sleep[Title/Abstract] OR Sleep, Inadequate[Title/Abstract] OR Sleep Debt[Title/Abstract] OR Sleep Insufficiency*[Title/Abstract] OR Insufficiency*, Sleep[Title/Abstract] OR Disorder*, Sleep Wake[Title/Abstract] OR Sleep Wake Disorder[Title/Abstract] OR Wake Disorder*, Sleep[Title/Abstract] OR Sleep Disorder*[Title/Abstract] OR Disorder*, Sleep[Title/Abstract] OR Long Sleeper Syndrome*[Title/Abstract] OR Sleeper Syndrome*, Long[Title/Abstract] OR Syndrome*, Long Sleeper[Title/Abstract] OR Short Sleeper Syndrome*[Title/Abstract] OR Sleeper Syndrome*, Short[Title/Abstract] OR Syndrome*, Short Sleeper[Title/Abstract] OR Short Sleep Phenotype*[Title/Abstract] OR Phenotype*, Short Sleep[Title/Abstract] OR Sleep Phenotypes, Short[Title/Abstract] OR Dyssomnia[Title/Abstract] OR Sleep Disorder*, Extrinsic[Title/Abstract] OR Extrinsic Sleep Disorder*[Title/Abstract] OR Adjustment Sleep Disorder*[Title/Abstract] OR Sleep Disorder*, Adjustment[Title/Abstract] OR Sleep Disturbance*[Title/Abstract] OR Somnipathy[Title/Abstract] OR Sleep Problem*[Title/Abstract] OR Sleep Issue*[Title/Abstract] OR Sleep Disruption[Title/Abstract] OR Sleep Dysfunction[Title/Abstract] OR Sleep Difficult*[Title/Abstract] OR Sleep Deprivation[Title/Abstract] OR Restless Sleep[Title/Abstract] OR Sleep restless*[Title/Abstract] OR Delayed Sleep Phase Syndrome[Title/Abstract] OR Delayed Sleep-Phase Syndrome*[Title/Abstract] OR Non-24 Hour Sleep-Wake Disorder[Title/Abstract] OR Non 24 Hour Sleep Wake Disorder[Title/Abstract] OR Sleep-Wake Disorder, Non-24 Hour[Title/Abstract] OR Sleep Wake Disorder, Non 24 Hour[Title/Abstract] OR Nonorganic Sleep Wake Cycle Disorder*[Title/Abstract]) AND (2004:2024[pdat]))** | 140499 |
| 5. | #3 AND #4  **(("Osteoarthritis"[Mesh] OR "Osteoarthritis, Knee"[Mesh] OR "Osteoarthritis, Hip"[Mesh]) OR (Osteoarthritides[Title/Abstract] OR Arthriti*, Degenerative[Title/Abstract] OR Degenerative Arthriti*[Title/Abstract] OR Osteoarthros*[Title/Abstract] OR Osteoarthrosis Deformans[Title/Abstract] OR Arthros*[Title/Abstract] OR Knee Osteoarthriti*[Title/Abstract] OR Osteoarthritis of the Knee[Title/Abstract] OR Osteoarthritis of Knee[Title/Abstract] OR Hip Osteoarthritis[Title/Abstract] OR Osteoarthritis of the Hip[Title/Abstract] OR Coxarthros*[Title/Abstract] OR Osteoarthritis Of Hip*[Title/Abstract] OR Wear-and-tear Arthritis[Title/Abstract] OR Joint degeneration[Title/Abstract] OR Cartilage Degradation[Title/Abstract] OR Bone Spur*[Title/Abstract] OR Spur*, Bone[Title/Abstract] OR Osteophyt*[Title/Abstract] OR Joint Stiffness[Title/Abstract] OR Joint Inflammation[Title/Abstract] OR Joint Effusion[Title/Abstract] OR Hydrarthroses[Title/Abstract]) AND (2004:2024[pdat])) AND ((("Sleep Quality"[Mesh] OR "Sleep Hygiene"[Mesh]) OR (Sleep qualit*[Title/Abstract] OR Qualit*, Sleep[Title/Abstract] OR Sleeping Habit*[Title/Abstract] OR Sleep Habit*[Title/Abstract] OR Habit*, Sleep[Title/Abstract] OR Habit*, Sleep*[Title/Abstract] OR Hygiene, Sleep[Title/Abstract] OR Good Sleep Habit*[Title/Abstract] OR Habit*, Good Sleep[Title/Abstract] OR Sleep Habit*, Good[Title/Abstract] OR Duration, Sleep[Title/Abstract] OR Sleep Quantity*[Title/Abstract] OR Quantity, Sleep[Title/Abstract] OR Total Sleep Time[Title/Abstract] OR Latency, Sleep[Title/Abstract] OR Sleep Latenc*[Title/Abstract] OR Sleep Onset Latenc*[Title/Abstract] OR Latency, Sleep Onset[Title/Abstract] OR Onset Latencies, Sleep[Title/Abstract] OR Sleep Pattern[Title/Abstract] OR Sleep Depth[Title/Abstract] OR Sleep Consistency[Title/Abstract] OR Sleep Efficiency[Title/Abstract] OR Sleep Satisfaction[Title/Abstract] OR Sleep Architecture[Title/Abstract] OR Restfulness[Title/Abstract] OR Sleep Soundness[Title/Abstract] OR Sleep Wellness[Title/Abstract] OR Sleep Health[Title/Abstract] OR Sleep State[Title/Abstract] OR Sleep Status[Title/Abstract] OR Sleep Continuity[Title/Abstract] OR Sleep Regularity[Title/Abstract] OR Sleep Variabilit*[Title/Abstract] OR Sleep Maintenance[Title/Abstract] OR Sleep Hygiene[Title/Abstract] OR Sleep fragmentation[Title/Abstract]) AND (2004:2024[pdat])) OR (("Sleep Initiation and Maintenance Disorders"[Mesh] OR "Sleep Wake Disorders"[Mesh] OR "Dyssomnias" [Mesh]) OR (Intrinsic Sleep Disorder*[Title/Abstract] OR Sleep Disorder, Intrinsic*[Title/Abstract] OR Sleep State Misperception*[Title/Abstract] OR Hypersomnia*, Post-Traumatic[Title/Abstract] OR Hypersomnia, Post Traumatic[Title/Abstract] OR Hypersomnias, Post-Traumatic Post-Traumatic Hypersomnia*[Title/Abstract] OR Hypersomnia*, Posttraumatic[Title/Abstract] OR Posttraumatic Hypersomnia*[Title/Abstract] OR Disorders of Initiating[Title/Abstract] AND Maintaining Sleep[Title/Abstract] OR Sleeplessness[Title/Abstract] OR Insomnia Disorder*[Title/Abstract] OR Insomnia*[Title/Abstract] OR Chronic Insomnia[Title/Abstract] OR Insomnia, Chronic[Title/Abstract] OR Early Awakening[Title/Abstract] OR Awakening, Early[Title/Abstract] OR Nonorganic Insomnia[Title/Abstract] OR Insomnia, Nonorganic[Title/Abstract] OR Primary Insomnia[Title/Abstract] OR Insomnia, Primary[Title/Abstract] OR Psychophysiological Insomnia[Title/Abstract] OR Insomnia, Psychophysiological[Title/Abstract] OR Rebound Insomnia[Title/Abstract] OR Insomnia, Rebound[Title/Abstract] OR Secondary Insomnia[Title/Abstract] OR Insomnia, Secondary[Title/Abstract] OR Sleep Initiation Dysfunction[Title/Abstract] OR Dysfunction*, Sleep Initiation[Title/Abstract] OR Sleep Initiation Dysfunctions[Title/Abstract] OR Transient Insomnia[Title/Abstract] OR Insomnia, Transient[Title/Abstract] OR Parasomnia[Title/Abstract] OR Sensory Paroxysm*, Sleep[Title/Abstract] OR Paroxysm*, Sleep Sensory[Title/Abstract] OR Sleep Sensory Paroxysm*[Title/Abstract] OR Deprivation, Sleep[Title/Abstract] OR Insufficient Sleep Syndrome*[Title/Abstract] OR Syndrome, Insufficient Sleep[Title/Abstract] OR REM Sleep Deprivation[Title/Abstract] OR Deprivation, REM Sleep[Title/Abstract] OR Sleep Deprivation, REM[Title/Abstract] OR Sleep Fragmentation[Title/Abstract] OR Fragmentation, Sleep[Title/Abstract] OR Insufficient Sleep[Title/Abstract] OR Sleep, Insufficient[Title/Abstract] OR Inadequate Sleep[Title/Abstract] OR Sleep, Inadequate[Title/Abstract] OR Sleep Debt[Title/Abstract] OR Sleep Insufficiency*[Title/Abstract] OR Insufficiency*, Sleep[Title/Abstract] OR Disorder*, Sleep Wake[Title/Abstract] OR Sleep Wake Disorder[Title/Abstract] OR Wake Disorder*, Sleep[Title/Abstract] OR Sleep Disorder*[Title/Abstract] OR Disorder*, Sleep[Title/Abstract] OR Long Sleeper Syndrome*[Title/Abstract] OR Sleeper Syndrome*, Long[Title/Abstract] OR Syndrome*, Long Sleeper[Title/Abstract] OR Short Sleeper Syndrome*[Title/Abstract] OR Sleeper Syndrome*, Short[Title/Abstract] OR Syndrome*, Short Sleeper[Title/Abstract] OR Short Sleep Phenotype*[Title/Abstract] OR Phenotype*, Short Sleep[Title/Abstract] OR Sleep Phenotypes, Short[Title/Abstract] OR Dyssomnia[Title/Abstract] OR Sleep Disorder*, Extrinsic[Title/Abstract] OR Extrinsic Sleep Disorder*[Title/Abstract] OR Adjustment Sleep Disorder*[Title/Abstract] OR Sleep Disorder*, Adjustment[Title/Abstract] OR Sleep Disturbance*[Title/Abstract] OR Somnipathy[Title/Abstract] OR Sleep Problem*[Title/Abstract] OR Sleep Issue*[Title/Abstract] OR Sleep Disruption[Title/Abstract] OR Sleep Dysfunction[Title/Abstract] OR Sleep Difficult*[Title/Abstract] OR Sleep Deprivation[Title/Abstract] OR Restless Sleep[Title/Abstract] OR Sleep restless*[Title/Abstract] OR Delayed Sleep Phase Syndrome[Title/Abstract] OR Delayed Sleep-Phase Syndrome*[Title/Abstract] OR Non-24 Hour Sleep-Wake Disorder[Title/Abstract] OR Non 24 Hour Sleep Wake Disorder[Title/Abstract] OR Sleep-Wake Disorder, Non-24 Hour[Title/Abstract] OR Sleep Wake Disorder, Non 24 Hour[Title/Abstract] OR Nonorganic Sleep Wake Cycle Disorder*[Title/Abstract]) AND (2004:2024[pdat])))** | 455 |
