# Supplementary material for: Sleep disturbances and sleep quality among individuals diagnosed with osteoarthritis: a systematic review and meta-analysis
Source: Front Med (Lausanne). 2025 Nov 19;12:1653047. doi: 10.3389/fmed.2025.1653047 (PMC12672537; doi:10.3389/fmed.2025.1653047)
Supplement: Supplementary file 2 [file Table_2.docx]

**Supplementary Table 2a. Quality appraisal to assess the risk of bias for cross-sectional studies**

| **Study (author & year)** | **1. Were the criteria for inclusion in the sample clearly defined?** | **2. Were the study subjects and the setting described in detail?** | **3. Was the exposure measured in a valid and reliable way?** | **4. Were objective, standard criteria used for measurement of the condition?** | **5. Were confounding factors identified?** | **6. Were strategies to deal with confounding factors stated?** | **7. Were the outcomes measured in a valid and reliable way?** | **8. Was appropriate statistical analysis used?** | **Total score out of 8** |
| --- | --- | --- | --- | --- | --- | --- | --- | --- | --- |
| Kakazu et al., 2024 | Yes | Yes | Yes | Yes | No | No | Yes | Yes | 6 |
| Garcia et al., 2015 | Yes | Yes | Yes | Yes | No | No | Yes | Yes | 6 |
| Cho et al., 2020 | Yes | Yes | Yes | Yes | Yes | Yes | Yes | Yes | 8 |
| Wang et al., 2023 | Yes | Yes | Yes | Yes | Yes | Yes | Yes | Yes | 8 |
| Tubanur Yilmaz et al., 2021 | Yes | Yes | Yes | Yes | No | No | Yes | Yes | 6 |
| Allen et al., 2016 | Yes | Yes | Yes | Yes | Yes | Yes | Yes | Yes | 8 |
| Afşar et al., 2018 | Yes | Yes | Yes | Yes | Yes | Yes | Yes | Yes | 8 |
| Park et al., 2019 | Yes | Yes | Yes | Yes | Yes | Yes | No | Yes | 7 |
| Kiyak et al., 2018 | Yes | Yes | Unclear | Yes | No | No | Yes | Yes | 5 |
| Martinez et al., 2019 | Yes | Yes | Yes | Yes | Yes | Yes | Yes | Yes | 8 |
| Jung et al., 2018 | Yes | Yes | Yes | Yes | Yes | Yes | No | Yes | 7 |
| Lapane et al., 2021 | Yes | Yes | Yes | Yes | Yes | Yes | Yes | Yes | 8 |
| Richard Oluyinka Akintayo, Abubakar Yerima, Courage Uhunmwangho, et al., 2019 | Yes | Yes | Yes | Yes | Yes | Yes | Yes | Yes | 8 |
| Feehan et al., 2020 | Yes | Yes | No | Yes | Yes | Yes | No | Yes | 6 |

**Supplementary Table 2b. Quality appraisal to assess the risk of bias for case-control studies**

| **Study (author & year)** | **1. Were the groups comparable other than the presence of disease in cases or the absence of disease in controls?** | **2. Were cases and controls matched appropriately?** | **3. Were the same criteria used for identification of cases and controls?** | **4. Was exposure measured in a standard, valid and reliable way?** | **5. Was exposure measured in the same way for cases and controls?** | **6. Were confounding factors identified?** | **7. Were strategies to deal with confounding factors stated?** | **8. Were outcomes assessed in a standard, valid and reliable way for cases and controls?** | **9. Was the exposure period of interest long enough to be meaningful?** | **10. Was appropriate statistical analysis used?** | **Total score out of 10** |
| --- | --- | --- | --- | --- | --- | --- | --- | --- | --- | --- | --- |
| Jacob et al., 2021 | Yes | Yes | Yes | Yes | Yes | Yes | Yes | Yes | Yes | Yes | 10 |

**Supplementary Table 2c. Quality appraisal to assess the risk of bias for cohort studies**

| **Study (author & year)** | **1. Were the two groups similar and recruited from the same population?** | **2. Were the exposures measured similarly to assign people to both exposed and unexposed groups?** | **3. Was the exposure measured in a valid and reliable way?** | **4. Were confounding factors identified?** | **5. Were strategies to deal with confounding factors stated?** | **6. Were the groups/participants free of the outcome at the start of the study (or at the moment of exposure)?** | **7. Were the outcomes measured in a valid and reliable way?** | **8. Was the follow up time reported and sufficient to be long enough for outcomes to occur?** | **9. Was follow up complete, and if not, were the reasons to loss to follow up described and explored?** | **10. Were strategies to address incomplete follow up utilized?** | **11. Was appropriate statistical analysis used?** | **Total score out of 11** |
| --- | --- | --- | --- | --- | --- | --- | --- | --- | --- | --- | --- | --- |
| Sasaki et al, 2014 | Yes | Yes | Yes | Yes | Yes | Unclear | Yes | Yes | No | No | Yes | 8 |
| Fu et al., 2019 | Yes | Yes | Yes | Yes | Yes | Yes | Yes | Yes | Yes | Yes | Yes | 11 |
